# Dynamic Path Planning of Mobile Robot Based on Improved Sparrow Search Algorithm

**DOI:** 10.3390/biomimetics8020182

**Published:** 2023-04-27

**Authors:** Lisang Liu, Jingrun Liang, Kaiqi Guo, Chengyang Ke, Dongwei He, Jian Chen

**Affiliations:** 1 School of Electronic, Electrical Engineering and Physics, Fujian University of Technology, Fuzhou 350118, China; 2 Fujian Province Industrial Integrated Automation Industry Technology Development Base, Fuzhou 350118, China

**Keywords:** sparrow search algorithm, Cauchy reverse learning, sine–cosine algorithm, Lévy flight strategy, dynamic window approach, path planning

## Abstract

Aiming at the shortcomings of the traditional sparrow search algorithm (SSA) in path planning, such as its high time-consumption, long path length, it being easy to collide with static obstacles and its inability to avoid dynamic obstacles, this paper proposes a new improved SSA based on multi-strategies. Firstly, Cauchy reverse learning was used to initialize the sparrow population to avoid a premature convergence of the algorithm. Secondly, the sine–cosine algorithm was used to update the producers’ position of the sparrow population and balance the global search and local exploration capabilities of the algorithm. Then, a Lévy flight strategy was used to update the scroungers’ position to avoid the algorithm falling into the local optimum. Finally, the improved SSA and dynamic window approach (DWA) were combined to enhance the local obstacle avoidance ability of the algorithm. The proposed novel algorithm is named ISSA-DWA. Compared with the traditional SSA, the path length, path turning times and execution time planned by the ISSA-DWA are reduced by 13.42%, 63.02% and 51.35%, respectively, and the path smoothness is improved by 62.29%. The experimental results show that the ISSA-DWA proposed in this paper can not only solve the shortcomings of the SSA but can also plan a highly smooth path safely and efficiently in the complex dynamic obstacle environment.

## 1. Introduction

Path planning is one of the key technologies for the autonomous navigation of mobile robot, which has been widely used in the fields of intelligent warehousing and logistics, autonomous driving and aerospace. The definition of path planning is that the mobile robot designs the route from the starting point to the end point according to the specified performance index to avoid collision with obstacles in a challenging and uncertain environment [1]. Depending on the degree of knowledge of environmental information, the path planning algorithm of mobile robot may be separated into global path planning and local path planning. The traditional global path planning algorithms, including the Dijkstra algorithm [2], A-star algorithm [3], RRT algorithm [4] and PRM algorithm [5], and the classical local path planning algorithms, such as the artificial potential field technique [6] and dynamic window approach [7], have all been proposed consecutively. Among the global path planning algorithms, scholars have made some achievements in path planning problems. In order to solve the problem of the vibration fatigue damage of large precision instruments in road transportation, Huang et al. [8] established a fatigue prediction model of equipment and used the Dijkstra algorithm to plan a low-risk path that can effectively reduce the vibration fatigue of equipment; however, the path planned by the algorithm is not smooth enough. Ju et al. [9] enhanced the A-star algorithm to provide the shortest path in the 2D static obstacle map, but the algorithm cannot avoid dynamic obstacles in real time. In order to shorten the path length and reduce the number of turns, Zhang et al. [10] suggested an enhanced A-star method based on adaptive neighborhood search and turning cost techniques. Wang et al. [11] proposed an enhanced RRT* algorithm for the path planning of mining trucks. To implement the route planning and gap driving of the pipeline environment and maintain a safe distance, Kamil et al. [12] revised the sampling method of the PRM algorithm. Among some local path planning algorithms, Liang et al. [13] improved the artificial potential field approach to plan the route of the UAV, which solved the problem of the UAV not being able to reach the target site. In another study, Li et al. [14] updated the velocity assessment function of the DWA and integrated it with the A-Star algorithm to avoid the nearby obstructions and plan a smoother path. However, the majority of these traditional global and local path planning methods are deterministic algorithms. Although they offer the benefits of simple principles, they also have the drawbacks of taking a long time, using a large amount of memory and being prone to local optimum solutions in challenging situations.

Scholars’ interest in some heuristic algorithms has recently increased. There are many classical heuristic algorithms used for the solution of the path planning, such as the particle swarm optimization algorithm (PSO) [15], ant colony optimization algorithm (ACO) [16], grey wolf optimization algorithm (GWO) [17], manta ray foraging optimization algorithm (MRFO) [18] and whale optimization algorithm (WOA) [19]. The classical heuristic algorithm has achieved certain results in solving the optimal path planning problem of mobile robot. Li et al. [20] used the cylindrical B-spline algorithm to plan the parallel manipulator’s trajectory in joint space, and the planned route was then further optimized by using the PSO algorithm; however, it is prone to local optimums and it is challenging for it to reach an accurate optimum solution during the iterative process. Wang et al. [21] combined the ACO algorithm with the DWA algorithm, which improved the path tracking accuracy of robot and realized the cooperative obstacle avoidance of multiple robots in an unknown environment. By integrating the bidirectional search with the ACO algorithm, Chen et al. [22] improved the A-star approach to boost the efficiency of path planning for mobile robot; however, the proposed approach has the drawbacks of a sluggish convergence speed and poor generalization ability. Yu et al. [23] combined the improved GWO algorithm with the D* Lite algorithm to realize the path planning of an unmanned cruise ship in an unknown obstacle environment. Nevertheless, the proposed algorithm has drawbacks, including it being easy for it to fall into local optimums and a lack of precision in the solutions. Zhou et al. [24] combined the GWO algorithm with the WOA algorithm for the global path planning of multi-robots, and fused the DWA for dynamic obstacle avoidance. Nonetheless, the algorithm is too complex and the path planning efficiency is not high.

With the intensive research of scholars, some new intelligence heuristic optimization algorithms have emerged. The sparrow search algorithm (SSA), as a brand-new meta-heuristic algorithm first put forward by Xue [25] in 2020, is mainly motivated by the sparrows’ foraging and anti-predator behavior, which has a higher convergence speed and a greater capacity for optimization on benchmark functions when compared to other classical algorithms. With the improvement in its performance, it is widely used in various research fields, such as path planning [26], the voltage regulation of a distribution network [27], battery health prediction [28], micro-grid operations planning [29] and so on. Nonetheless, similar to other swarm intelligence optimization techniques, the population diversity of the SSA will progressively decline in subsequent iterations, and it is simple for it to enter a local optimum. Yang et al. [30] used a chaotic mapping method to initialize the sparrow population, which increased the diversity of the population and improved the search ability of the algorithm, but the convergence speed of the algorithm was not accelerated. Yu et al. [31] proposed a sparrow particle swarm algorithm (SPSA) for UAV path planning that can significantly reduce blind search and improve the smoothness of the UAV flight trajectory. Zhang et al. [32] used reverse learning theory to improve the sparrow algorithm and applied it to the path planning problem of mobile robot. Ouyang et al. [33] proposed a learning sparrow search algorithm (LSSA) that uses the random reverse learning theory in the search stage of the SSA algorithm to enrich the diversity of the sparrows’ population. To solve the problem of the dynamic obstacle avoidance of multi-UAVs in a mountainous environment, Liu et al. [34] proposed a hybrid algorithm based on the SSA and a bioinspired neural network (BINN). The simulation results show that the proposed algorithm can plan a smooth collision-free shortest path in a mountainous environment. Li et al. [35] employed the simulated annealing technique to force the SSA algorithm to leave the local optimum throughout the iterative process, but the speed of the convergence is too sluggish and the accuracy of the solution still has to be increased. By utilizing a linear path approach and a novel domain search method, Zhang et al. [36] used the improved SSA to reduce the convergence time and increase the smoothness of the path. Although the proposed method provided a global planning path, it has drawbacks, including the path not being smooth enough and it not being able to avoid dynamic obstacles.

In line with the above research, this paper focused on the innovative improvement and application of the SSA. When the literature is examined, studies combining the improved SSA with the DWA to solve the optimal path problem of the mobile robot have not been found. Aiming at the shortcomings of the SSA, this paper proposes an improved SSA based on Cauchy reverse learning, the sine-cosine algorithm, the Lévy flight strategy and the DWA algorithm, which is called the ISSA-DWA algorithm. Firstly, the Cauchy reverse learning technique was used to initialize the sparrow population, which enriches the diversity of sparrow population, avoids the premature convergence of the algorithm due to an uneven population distribution in the later iteration and improves the efficiency of path planning. Secondly, the sine–cosine algorithm was used to update the producers’ position of the sparrow population, which balances the exploration and exploitation ability of the algorithm. Then, the Lévy flight strategy was adopted to update the position of scroungers so as to optimally improve the ability of the algorithm to jump out of the local optimum. Finally, the improved DWA was used for the local path planning and dynamic obstacle avoidance of mobile robot. By improving the azimuth evaluation function of the DWA, fusing the optimal path point information given by the ISSA and setting temporary sub-target points, the mobile robot can not only move smoothly along the global optimal path planned by the ISSA but can also avoid the dynamic obstacle in real time.

The main contributions of this paper are as follows:

1. A novel swarm-based algorithm for solving the optimal path planning problem of the mobile robot, named as ISSA-DWA, is proposed by combining several advanced strategies in this paper.
2. In the proposed ISSA-DWA method, the Cauchy reverse learning theory is used to enrich the diversity of sparrow population to avoid the algorithm falling into premature maturity.
3. In order to solve the problem of the weak search ability in the later iterations of the SSA, the sine–cosine algorithm is used to update the location of the producers so as to balance the exploration and exploitation capability of the algorithm.
4. Since the SSA is prone to fall into local optimal solutions, the Lévy flight strategy is used to update the scroungers’ position in the ISSA-DWA, which could increase the probability of jumping out of the local optimum.
5. An improved azimuth evaluation function is proposed in the ISSA-DWA. Combined with the optimal path point information given by the ISSA, the proposed ISSA-DWA makes the mobile robot move smoothly to the optimal path.
6. The experimental results and analysis show that the proposed ISSA-DWA is more competitive in solving the optimal path planning problem of mobile robot.

The rest of this paper is organized as follows: the standard SSA introduction is given in Section 2. Then, an improved SSA with multi-strategies is introduced in Section 3 in detail. Section 4 introduces the standard DWA and its improvement. Section 5 presents the experimental results of the proposed ISSA-DWA and compares the performance advantages with the classic algorithms. Finally, Section 6 summarizes the study and highlights the future work.

## 2. Standard Sparrow Search Algorithm

The standard SSA is a brand-new swarm intelligence optimization technique that mimics the predation and anti-predation of sparrows. According to Ref. [25], it has the benefits of a rapid convergence and high convergence accuracy, but it also has the drawbacks of insufficient population diversity and easy to fall into local optimum.

It is assumed that the sparrow set matrix is:

(1) $$X={\left[x_{1},x_{2},x_{3},\ldots ,x_{n}\right]}^{T},x_{i}=[x_{i,1},x_{i,2},\ldots ,x_{i,d}]$$

where *n* is the number of sparrows, *i* = (1,2,…,*n*) and *d* is the sparrows’ dimension.

According to Formula (1), the sparrow’s fitness function can be expressed as Equation (2):

(2) $$F(x)={\left[f(x_{1}),f(x_{2}),\ldots ,f(x_{n})\right]}^{T},f(x_{i})=[f(x_{i,1}),f(x_{i,2}),\ldots ,f(x_{i,d})]$$

Here, the fitness score of the *i*-th sparrow is represented by *f(x_i_)*.

In the standard SSA, the whole population will move to the food source, and the sparrows with priority access to food and good fitness will become producers. The producers’ location is updated as follows:

(3) $$X^{t+1}{{}_{i}}_{,j}=\{\begin{array}{c} X^{t}{{}_{i}}_{,j}\cdot \exp(\frac{-i}{\partial \cdot iter_{\max}}),R_{2}<ST\\ X^{t}{{}_{i}}_{,j}+\Phi \cdot B,R_{2}\geq ST \end{array}$$

where *t* represents the quantity of population iterations, *j* = (1, 2, …, *d*), $X_{i,j}^{t}$ denotes the location of the *i*-th sparrow in the *j*-th dimension, *iter*_max_ indicates the max iterations and ∂∈(0,1) displays the random numbers. The safety value is represented by ST∈[0.5,1], whereas the warning value is represented by $R_{2}\in [0,1]$. A random integer with a normal distribution from 0 to 1 is designated by the symbol Φ. *B* represents the 1×d matrix in which the value of each element is 1. When $R_{2}<ST$, it indicates that there are no natural adversaries around the sparrows, producers are free to hunt for food in a variety of locations. Otherwise, if $R_{2}\geq ST$, it indicates that some sparrows have discovered natural enemies and that the entire colony must immediately fly to other safe regions. It is worth noting that, when $R_{2}\geq ST$, the dimension of the producer will decrease slowly in the later stage of iterations, which will lead to problems such as an insufficient search space ability and the poor convergence speed of the algorithm.

Except for the producers, most of the remaining sparrows are scroungers. The scroungers’ position is updated as follows:

(4) $$X^{t+1}{{}_{i}}_{,j}=\{\begin{array}{c} \Phi \cdot \exp(\frac{X^{t}worst-X^{t}{{}_{i}}_{,j}}{i^{2}}),i>\frac{n}{2}\\ X_{p}^{t+1}+|X^{t}{{}_{i}}_{,j}-X_{p}^{t+1}|\cdot Z^{\prime }\cdot B,i\leq \frac{n}{2} \end{array}$$

where $X_{worst}$ stands for the sparrow individual with the worst fitness value globally. $X_{p}^{t+1}$ denotes the best position of the producers. *Z* is the 1×d matrix whose values are allocated at random to either 1 or −1, and $Z^{\prime }=Z^{T}{\left(ZZ^{T}\right)}^{-1}$. When i>n/2, it signifies that the *i*-th scroungers did not obtain food due to its poor fitness value. To increase its fitness value, the scroungers need to go to various locations to hunt for food.

Approximately 10–20% of the sparrow population will be randomly chosen as scouters to remain watchful of their surroundings throughout the foraging phase. All sparrows, producers and scroungers alike, must abandon their present food source and migrate to a new area once the sparrows identify its natural opponents. The scouters’ location is updated as follows:

(5) $$X^{t+1}{{}_{i}}_{,j}=\{\begin{array}{c} X^{t}{}_{best}+b\cdot |X^{t}{{}_{i}}_{,j}-X^{t}{}_{best}|,f_{i}>f_{g}\\ X^{t}{{}_{i}}_{,j}+k\cdot (\frac{\left|X^{t}{{}_{i}}_{,j}-X^{t}{}_{worst}\right|}{(f_{i}-f_{w})+\varepsilon }),f_{i}=f_{g} \end{array}$$

where $X_{best}$ stands for the best position of the sparrow individuals globally.b is a random value from 0 to 1, which follows a normal distribution and has a configurable step size. k∈[−1,1], which stands for a random number. The current global best and worst fitness values are represented by $f_{g}$ and $f_{w}$, respectively. The minimal constant used to prevent a numerator of zero is ε. If $f_{i}>f_{g}$, it denotes that it is not easy for the sparrows to be attacked by natural enemies. Otherwise, the sparrows are outside of or close to the colony, and are exposed to attacks by its enemies. *k* represents the direction of the sparrows’ movement.

**Figure 1:**
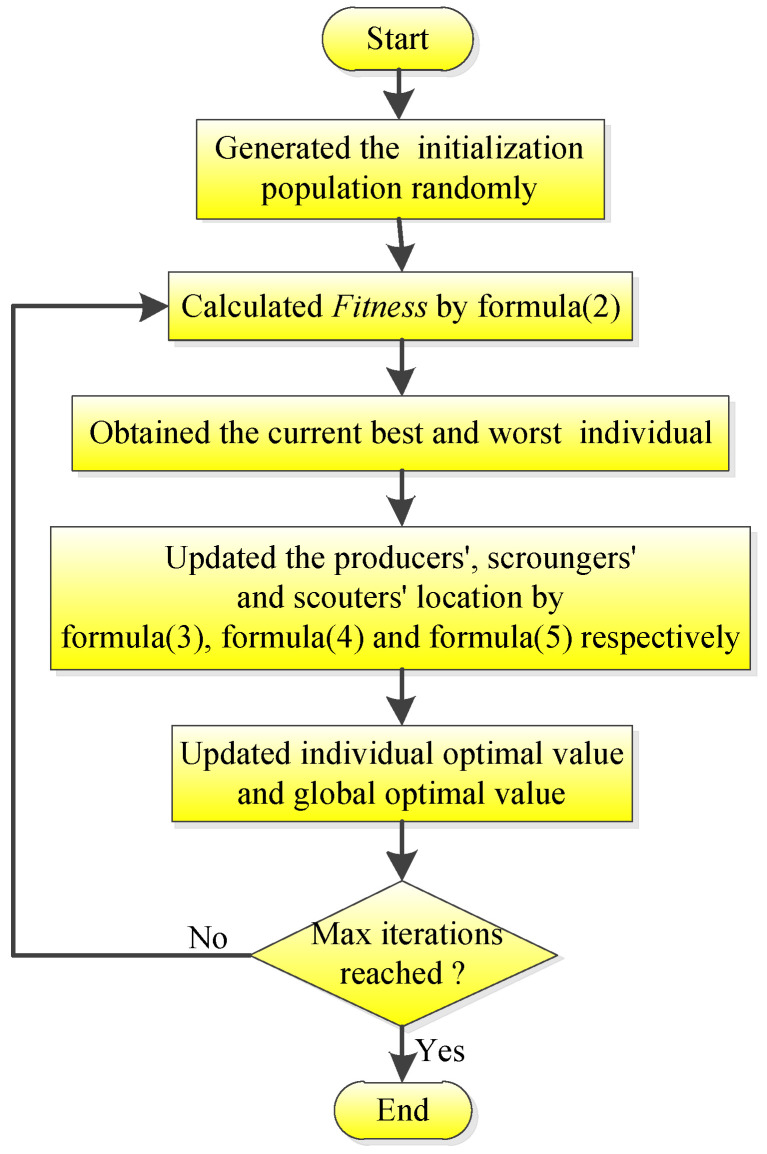
Flow chart of the standard SSA.

The flow chart for the standard SSA is shown in Figure 1.

In summary, the standard SSA has the following disadvantages:

1. The initial population of the standard SSA is generated at random, and there are several issues existing, including an uneven distribution of the population and poor diversity. In the subsequent iterations, this will lead to an insufficient search scope, low quality of the initial solution and slow convergence speed of the algorithm.
2. The update method of the producers’ location of the standard SSA is poor, where it is unable to balance both the exploration and exploitation capability. The dimensions of producers will decrease slowly in the later iteration, which will lead to a decline in its search ability and slow convergence speed.
3. Since the standard SSA algorithm iterates for a specific amount of times, if the fitness value of the producers stayed constant, the producers will become the scroungers and the algorithm will fall into the local optimum easily.

## 3. Improved Sparrow Search Algorithm

In view of the shortcomings of the standard SSA, this paper proposes the following methods to improve its performance:

1. Pointing at the disadvantages of the standard SSA, such as an insufficient population distribution and population diversity, Cauchy reverse learning was used to initialize the sparrow population, which enriches the diversity of the sparrow population and improves the quality of the initial solution of the algorithm.
2. Aiming at the problem of the poor update method of the producers’ location, the sine–cosine algorithm and dynamic learning factor were used to balance both the exploration and exploitation capability.
3. To solve the problem of it being easy for the standard SSA to fall into the local optimum, the Lévy flight strategy was used to update the position of the scroungers and increase the probability of the algorithm jumping out of the local optimal solution.

### 3.1. Cauchy Reverse Learning

Through the reverse learning mechanism, the Cauchy reverse learning algorithm can generate the reverse population of the initial population. Compared with the initial population, the better individuals will be chosen as the next generation population in the reverse population. The selected individuals are currently approaching the ideal solution than those in the reverse population and the initial population, which significantly accelerates the convergence of the algorithm. In addition, compared with the random initialization and chaotic mapping initialization, the initialization of the Cauchy reverse learning algorithm has several advantages, such as a large effective searching area, rich population variety and excellent global search ability. Therefore, this paper used the Cauchy reverse learning algorithm to establish the sparrow initialization population to address the issues of the uneven population distribution and poor population diversity of the standard SSA.

Assume that the point $P=x\left(x_{1},x_{2},x_{3},\ldots ,x_{d}\right)$ is located in a *d*-dimensional space, where $x_{i}\in [a_{i},b_{i}]$ and i = 1, 2, …, d. The lowest and highest values of point *P*’s *i*-th dimensional space are $a_{i}$ and $b_{i}$, respectively. Then, the inverse point of point *P* is:

(6) $$OP=x^{\prime }(x_{1}^{\prime },x_{2}^{\prime },x_{3}^{\prime },\ldots ,x_{d}^{\prime })$$

where $x_{i}^{\prime }=a_{i}+b_{i}-x_{i}$. Then, according to the definition of the Cauchy inverse point [37], it can be known that the Cauchy inverse point at point *P* is:

(7) $$OP_{c}^{\prime }=rand(\frac{a_{i}+b_{i}}{2},x_{i}^{\prime })$$

where *i* = 1, 2, …, *d*. The Cauchy reversal point is a randomly produced point that is between the middle point and the typical reversal point, as shown by Formula (7). As a result, in accordance with the definition of the Cauchy reverse point, the Cauchy reverse population of the initial population is created by using the Cauchy reverse learning algorithm. Then, the fitness values of the Cauchy reverse population and the initial population are calculated and compared. Finally, the individuals with good fitness values are added to the final initialization population of the sparrows.

### 3.2. Sine–Cosine Algorithm

The sine–cosine algorithm (SCA), a dynamic adjustment algorithm, can reduce algorithmic unpredictability in the later iterations of the algorithm [38]. When compared to other dynamic adjustment algorithms, such as fuzzy control, the SCA algorithm has the advantages of being highly adaptable, highly robust and flexibly responsive, which can further improve the global search ability of the algorithm and accelerate the convergence [39]. Therefore, by combining the SCA algorithm and the learning factor, the position of the producers can be updated and adjusted adaptively. In the initial stages of the algorithm, the SCA algorithm and the learning factor improves the algorithm’s performance in global search. In the later iterations, the SCA algorithm and the learning factor increases the ability of the local mining of the algorithm. Thus, the exploration and exploitation capability of the algorithm can be balanced by the SCA algorithm and the learning factor adaptively. The formula for the learning factor is proposed as follows:

(8) $$\lambda =\lambda_{\min}+\frac{\left(\lambda_{\max}-\lambda_{\min}\right)}{\left(\lambda_{\max}+\lambda_{\min}\right)}\cdot \cos(t\cdot \pi /{iter}_{max})$$

where λ is the learning factor. The learning factor’s minimal and maximal values are represented by $\lambda_{\min}$ and $\lambda_{\max}$, respectively. Other variables have been clarified in Formula (3), so they will not be described here again.

By combining the SCA algorithm with the learning factor, the improved producers’ location is updated as follows:

(9) $$X^{t+1}{{}_{i}}_{,j}=\left\{\begin{array}{c} (1-\lambda )\cdot X^{t}{{}_{i}}_{,j}+\lambda \cdot \sin(r_{1})\cdot |r_{2}\cdot X_{best}-X^{t}{{}_{i}}_{,j}|,R_{2}<ST\\ (1-\lambda )\cdot X^{t}{{}_{i}}_{,j}+\lambda \cdot \cos(r_{1})\cdot |r_{2}\cdot X_{best}-X^{t}{{}_{i}}_{,j}|,R_{2}\geq ST \end{array}\right.$$

where $r_{1}$ is a random value between [0,2π] and $r_{2}$ is a random value between [0,2]. Other variables have been clarified in Formulas (3) and (5), so they will not be described here again.

### 3.3. Lévy Flight Strategy

The Lévy flight strategy, which combines continuous small steps with sporadic big steps, prevents the algorithm from exploring the same area twice, broadens the algorithm’s search space and enhances its search ability [40]. Because of its characteristics, it can be used to address the issue of the standard SSA being vulnerable to entering the local optimum. As a result, this paper used the Lévy flight strategy to update the scroungers’ location, and the improved formula is expressed as follows:

(10) $$X^{t+1}{{}_{i}}_{,j}=\{\begin{array}{l} \Phi \cdot \exp(\frac{X^{t}{}_{worst}-X^{t}{{}_{i}}_{,j}}{i^{2}}),i>\frac{n}{2}\\ X_{p}^{t+1}+X_{p}^{t+1}⊗Lé\mathrm{vy}(d),i\leq \frac{n}{2} \end{array}$$

where the Lévy(*d*) stands for the moving distance of *d*-dimensional sparrows using the Lévy flight strategy. Other variables have been clarified in Formula (4), so they will not be described here again.

The formula for the Lévy flight strategy is expressed as follows:

(11) $$Lé\mathrm{vy}(x)=0.01\cdot \frac{r3\cdot \sigma }{\sqrt[{\zeta }]{\left|r4\right|}}$$

where both $r_{3}$ and $r_{4}$ are arbitrary values between 0 and 1. ζ is a constant. When taking ζ=1.5, the formula for calculating σ is described in the following:

(12) $$\sigma ={\left(\frac{\Gamma (1+\zeta )\cdot \sin(\pi \cdot \zeta /2)}{\Gamma ((1+\zeta )/2)\cdot \zeta \cdot 2^{(\zeta -1)/2}}\right)}^{1/\zeta }$$

Here, Γ(x)=(x−1)!.

### 3.4. Path Optimization Strategy

**Figure 2:**
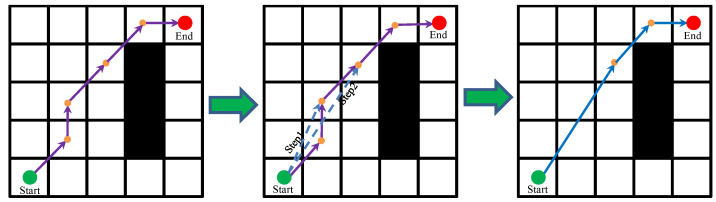
Iterative process of the ILPS strategy.

**Figure 3:**
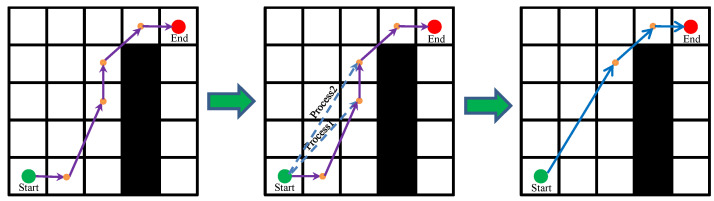
Iterative process of the INSS strategy.

For the convenience of modeling, we regard the mobile robot as a particle. In Ref. [41], a linear path strategy (LPS) was proposed, but it has the shortcoming of being time-consuming. In Ref. [36], an improved LPS strategy and neighborhood search strategy were proposed to improve the path quality and path acquisition time. However, it also has drawbacks, such as path node redundancy and it being easy for the fitness value to fall into the local optimum. Therefore, based on the LPS strategy and neighborhood search strategy, this paper further improved its algorithm structure and proposed the improved LPS strategy (ILPS) and improved neighborhood search strategy (INSS), which reduced the path node and the fitness value of the optimal individuals. Furthermore, the ILPS strategy was used to detect obstacles and optimize path corners. By calculating the fitness value of the current path, the path with a smaller fitness value is considered as the new path, and the position of the sparrow population is updated. The INSS strategy was used to further reduce the fitness value of the sparrow population on the basis of the global optimal individual. The iterative processes of the ILPS strategy and the INSS strategy are shown in Figure 2 and Figure 3, respectively.

In Figure 2 and Figure 3, the green circle represents the start point, red circle represents the end point and yellow points represent the path point. In addition, the purple solid line represents the path before optimization and the blue solid line represents the optimized path.

**Figure 4:**
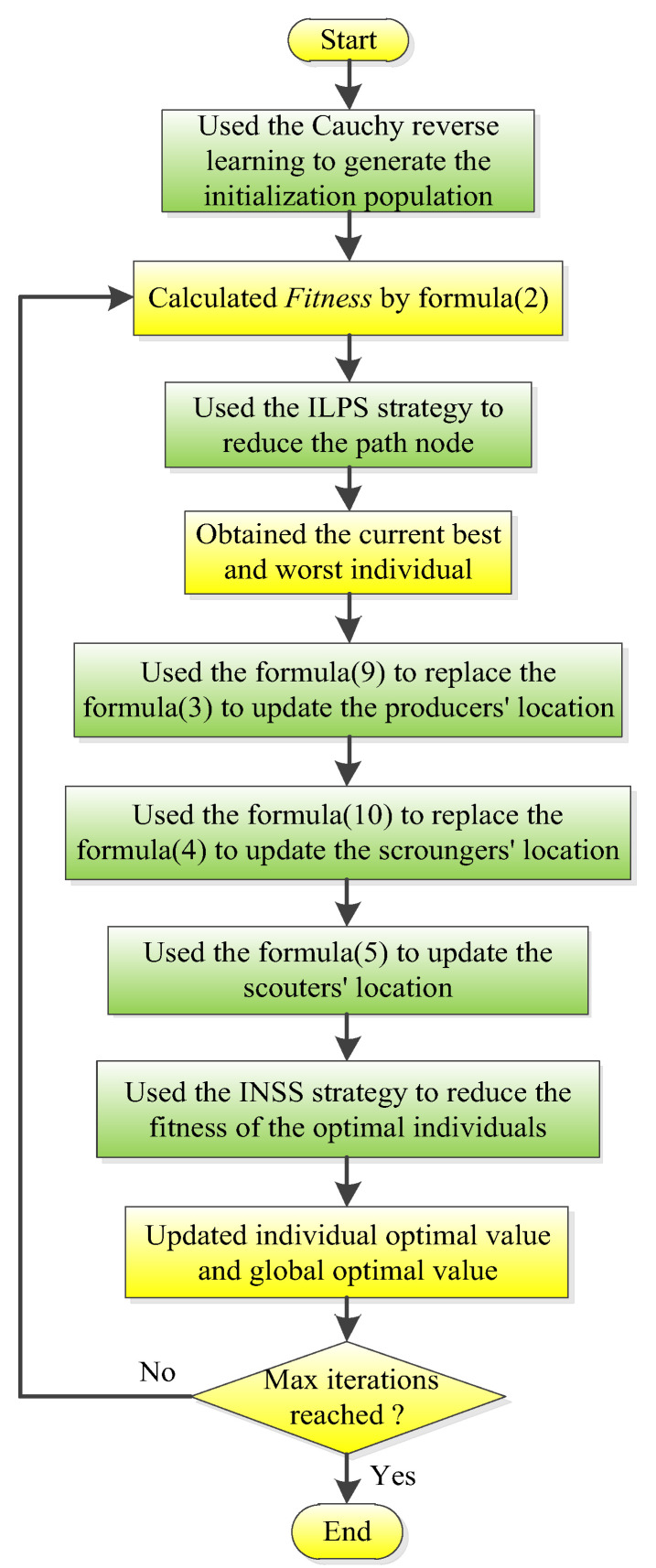
Flow chart of the ISSA algorithm.

As can be seen from the figure, the path length was changed significantly by adding the ILPS strategy and INSS strategy to the proposed ISSA algorithm. In Figure 2, after removing two path points using Step1 and Step2 of the ILPS strategy, the path length after ILPS strategy optimization is obviously shorter than that before. Similarly, in Figure 3, the path points are removed by Process1 and Process2 of the INSS strategy, which further reduces the fitness value of global optimal individuals and improves the probability of the algorithm jumping out of the local optimum. It is precisely because the ILPS strategy has an excellent path angle optimization ability and the INSS strategy can obviously reduce the optimal individual fitness value that these two strategies are considered as two vital components of the proposed ISSA algorithm. The flow chart of the proposed ISSA algorithm in this paper is shown in Figure 4.

## 4. Dynamic Window Approach and Its Improvements

### 4.1. Standard Dynamic Window Approach

The standard dynamic window approach has the advantages of real-time obstacle avoidance and a smooth route. In the velocity vector space composed of multiple velocities and accelerations, the DWA method samples multiple groups of velocities of the mobile robot model according to its own finite velocity and acceleration constraints, and deduces the motion trajectories of these velocities in a certain time interval. At this time, these trajectories can be scored according to certain evaluation indexes, and the trajectory with the highest score can be selected as the final trajectory of the mobile robot. The core of the DWA is to transform the local path planning problem into a constrained optimal solution problem in the velocity vector space by controlling and searching the velocity of the mobile robot [42].

#### 4.1.1. A Mobile Robot Model

To implement the DWA method, the mobile robot’s motion model must first be constructed. Assuming that the mobile robot cannot move in all directions, its motion trajectory is composed of each small arc, and the motion state of the small arc can be described by a two-dimensional velocity space (v,ω). Since the time interval Δt is very small, the mobile robot’s movement in Δt can be approximated as a uniform linear motion, and the robot’s motion model may be expressed as [43]:

(13) $$\{\begin{array}{c} x_{t}+1=x_{t}+v_{t}\Delta t\cos\theta_{t}\\ y_{t}+1=y_{t}+v_{t}\Delta t\sin\theta_{t}\\ \theta_{t}+1=\theta_{t}+\omega_{t}\Delta t \end{array}$$

where $\left(x_{t},y_{t}\right)$ and $\theta_{t}$ are the coordinates and the azimuth at moment *t* of the mobile robot, respectively. Similarly, $\left(x_{t+1},y_{t+1}\right)$ and $\theta_{t+1}$ are the coordinates and the azimuth at moment *t+*1 of the mobile robot, respectively. $v_{t}$ and $\omega_{t}$ are the linear and angular velocity at moment *t* of the mobile robot, respectively.

#### 4.1.2. Velocity Sampling

After the mobile robot model is established, it needs to be sampled in multiple groups to calculate the range of the sampling velocity of the mobile robot’s motion trajectory. The gamut of the sampling velocity is typically governed by the following three factors:

1. Restricted by the maximum and minimum velocity of the robot’s own model:

(14) $$V_{m}=\{(v,\omega )|v\in [v_{\min},v_{\max}],\omega \in [\omega_{\min},\omega_{\max}]\}$$

where $V_{m}$ indicates the maximum and minimum velocity space of the robot. The robot’s maximum and minimum linear velocities are represented by $v_{\max},v_{\min}$, respectively, and the robot’s maximum and minimum angular velocities are represented by $\omega_{\max},\omega_{\min}$, respectively.

2. Limited by the safe distance between the robot and the obstruction:

(15) $$V_{a}=\{(v,\omega )|v\leq \sqrt{2\cdot dist(v,\omega )\cdot vb}’\wedge \omega \leq \sqrt{2\cdot dist(v,\omega )\cdot \omega_{b}}’\}$$

where $V_{a}$ indicates the safe velocity space of the robot, dist(v,ω) represents the distance between the trajectory simulated by the velocity vector (v,ω) and the nearest obstacle. The maximal linear and angular decelerations of the robot are represented by $v_{b}^{’},\omega_{b}^{’}$, respectively.

3. Limited by the performance of the robot’s motors:

(16) $$V_{d}=\{(v,\omega )|v\in [v_{c}-v_{b}^{\prime }\cdot \Delta t,v_{c}+v_{a}^{\prime }\cdot \Delta t]\wedge \omega \in [\omega_{c}-\omega_{b}^{\prime }\cdot \Delta t,\omega_{c}+\omega_{a}^{\prime }\cdot \Delta t]\}$$

where $V_{d}$ indicates the maximum and minimum velocity space that the robot could reach. $v_{c},\omega_{c}$ represent the current linear velocity and angular velocity of the robot, respectively.$v_{a}^{\prime },\omega_{a}^{\prime }$ represent the maximum linear acceleration and the maximum angular acceleration of the robot, respectively.$v_{b}^{\prime },\omega_{b}^{\prime }$ represent the maximum linear deceleration and the maximum angular deceleration of the robot, respectively. 

#### 4.1.3. Evaluation Function

After velocity sampling, several feasible simulation trajectories can be calculated. In order to ensure that the mobile robot can reach the target position safely and effectively, it is necessary to evaluate these simulated trajectories in multiple dimensions and select the simulated trajectory with the highest score as the final trajectory of the mobile robot. There are three criteria for evaluating the simulated trajectory by the standard DWA: azimuth, obstacle distance and velocity. The overall evaluation function of the standard DWA can be expressed as:

(17) G(v,ω)=α⋅head(v,ω)+β⋅dist(v,ω)+γ⋅vel(v,ω)

where the weighting factors for the three evaluation indicators are denoted by α,β,γ, respectively. The evaluation functions for azimuth, obstacle distance and velocity are shown by head(v,ω),dist(v,ω),vel(v,ω), respectively. Furthermore, head(v,ω) describes the deviation angle of the target position of the robot and the end point of its trajectory at the current sampling velocity, dist(v,ω) describes the distance between a robot’s current trajectory and a nearby obstruction and vel(v,ω) describes the velocity of the current trajectory.

### 4.2. Improved Dynamic Window Approach

Since the azimuth evaluation function head(v,ω) of the standard DWA only evaluates the target point, it lacks the reference of the global optimal path information. When facing a complex obstacle environment, it is often easy for it to fall into the local optimum, and it may even fail to reach the destination. Therefore, by improving the azimuth function of the standard DWA, this paper proposed a new azimuth evaluation function, BPhead(v,ω), and added a smoothing function σ and smoothing factor φ so that the mobile robot can move along the global optimal path and have an excellent path smoothing ability and dynamic obstacle avoidance ability. The evaluation function of the improved DWA method is expressed as follows:

(18) G(v,ω)=φ⋅σ(α⋅BPhead(v,ω)+β⋅dist(v,ω)+γ⋅vel(v,ω))

where BPhead(v,ω) is the azimuth deviation function between the orientation of the end of the simulated trajectory and the global optimal path point nearest to the current trajectory. σ is the smoothing function. φ is the smoothing factor.

#### 4.2.1. Algorithm Fusion

After improving the DWA, it can then be combined with the ISSA to be a novel dynamic path planning fusion algorithm, which is called the ISSA-DWA algorithm. The ISSA-DWA algorithm proposed in this paper can not only solve the shortcomings of the standard SSA and standard DWA, but also the local dynamic obstacle avoidance ability and path smoothing ability of the algorithm are further improved. The pseudo code of the ISSA-DWA is as follows Algorithm 1:
**Algorithm 1:** ISSA-DWA pseudo code**Input:**Warning value: *R_2_.*Safety value: *ST*.The number of sparrow populations: *n*.The maximum number of iterations: *iter*_max_.The initial number of producers: *PDNumber*.The initial number of sparrows in charge of vigilance: *SDNumber*.The parameters of the DWA: α,β,γ,φ**Output:** The smooth optimal trajectory.1.Initialize the population of sparrows by applying Cauchy reverse learning to Formula (7);2.Choose the sparrow in the initial population with the lowest fitness value as the present best option;3.**While** (*t* < *iter*_max_)4.Use the ILPS strategy to reduce the path node;5.**for***i* = 1: *PDNumber*6.Update the location of the producers with the improved Formula (9);7.**end for**8.**for** *i* = (*PDNumber* + 1): *n*9.Update the location of the scroungers with the improved Formula (10);10.**end for**11.**for** *i* = 1: *SDNumber*12.Update the location of the sparrows with Formula (5);13.**end for**14.Obtain the current new location;15.Use the INSS strategy to reduce the fitness of the optimal individuals;16.Update the optimal location of the current individual;17.*t = t + 1*;18.**end While**19.Acquire global optimal path point information of the **ISSA**;20.Fuse the point information and put it into local planner DWA;21.Control the robot to move according to the global optimal path;22.**return** the smooth optimal trajectory.


#### 4.2.2. Complexity Analysis

The computational complexity of an algorithm can be described by the Big-O notation. When solving the optimal path planning problem of the mobile robot, the complexity relies on the number of sparrows (*n*), the dimensions of the path planning problem (*d*), the number of iterations (*iter*_max_) and the function assessment’s cost (*c*). In specific terms, the overall computational complexity of the proposed ISSA-DWA can be given as below:

(19) $$\begin{array}{ll} O\left(\mathrm{ISSA}-\mathrm{DWA}\right) & =O\left(path\;planning\;problem\right)+\;O\left(init.\right)\\ & +O\left(cost\;function\right)+O\left(solution\;update\right)\\ & =O\left(1+n\cdot d+iter_{\max}\cdot c\cdot n+iter_{\max}\cdot n\cdot d\right)\\ & ≅O\left(iter_{\max}\cdot c\cdot n+iter_{\max}\cdot n\cdot d\right) \end{array}$$

## 5. Experiments and Analysis

### 5.1. Experimental Environment Construction

**Figure 5:**
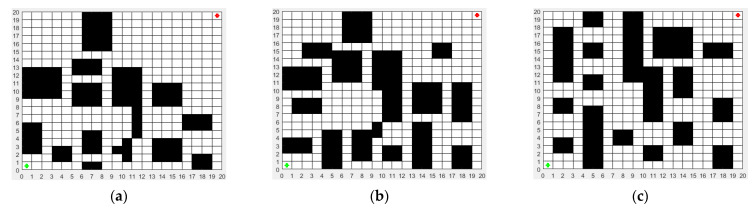
Three environmental models for path planning of the mobile robot: (**a**) environment model 1 (ENV. 1); (**b**) environment model 2 (ENV. 2); (**c**) environment model 3 (ENV. 3). The green dot represents the start point and the red dot represents the end point.

In order to better describe the real environment of the mobile robot, three environmental models of the mobile robot were established as shown in Figure 5. The map size of the three environmental models is 20 m × 20 m. The green dot represents the start point, and its coordinate is (1,1). The red dot represents the end point, and its coordinate is (20,20). By comparison, it can be seen that the environmental models in Figure 5b,c are more complex than that in Figure 5a, which will be a challenge for the path planning of the mobile robot.

In addition, the simulation environment of the experiment in this paper was a laptop with processor Intel(R) Core(TM) i7-5500 @ 2.40 GHz, memory 8 GB and Windows 7 64-bit system with MATLAB R2017b software.

### 5.2. Static Obstacle Avoidance Experiment

**Figure 6:**
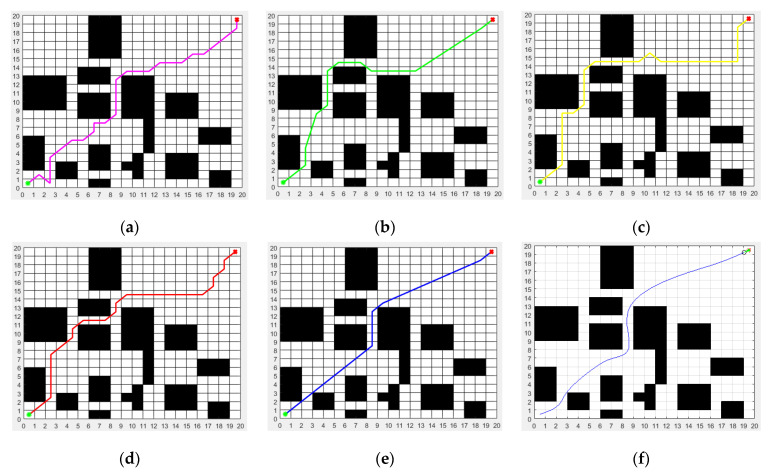
Path planned by the algorithms (ACO, MRFO, WOA, SSA, ISSA and ISSA-DWA) in environment model 1: (**a**) path planned by ACO; (**b**) path planned by MRFO; (**c**) path planned by WOA; (**d**) path planned by SSA; (**e**) path planned by ISSA; (**f**) path planned by ISSA-DWA. The green dot represents the start point and the red dot represents the end point.

It was assumed that the mobile robot can be regarded as a point and that it can only move in the two-dimensional grid area. In the static obstacle avoidance simulation experiment, this paper set up three groups of comparative experiments for three different map environment models. Since the proposed ISSA-DWA algorithm increases its path smoothing ability and dynamic obstacle avoidance ability on the basis of the proposed ISSA algorithm, the global path planning ability is not affected. The static obstacle avoidance simulation experiment actually tests the static characteristics of the path planning algorithm. When only the path length, path turning times, path smoothness and execution time of the algorithm are considered as the measurement criteria, the static obstacle avoidance effect of the proposed ISSA-DWA algorithm can be regarded as equivalent to that of the proposed ISSA algorithm. Therefore, in the static obstacle avoidance simulation experiment, we only needed to compare the proposed ISSA algorithm with four basic heuristic algorithms, such as the ACO, MRFO, WOA and SSA algorithm. To obtain a fair and valid comparison of the results, all experiments were conducted on the same computer. The population size for both the proposed ISSA and the other four basic heuristic algorithms was set to 50, and the maximum number of iterations was set to 200. In environmental model 1, the ACO, MRFO, WOA, SSA and ISSA algorithms were applied to the static path planning of the mobile robot, and the ISSA-DWA algorithm was applied to the dynamic path planning of the mobile robot. The experimental simulation results of the first group are shown in Figure 6.

Figure 6a–e show the static path planning and static obstacle avoidance effects of the ACO, MRFO, WOA, SSA and ISSA in environmental model 1, respectively, and Figure 6f shows the dynamic path planning and static obstacle avoidance effects of the proposed ISSA-DWA algorithm in environmental model 1. When only the static path planning effect is considered, it is not difficult to see from the figure that the proposed ISSA algorithm has a shorter path length, fewer path turning times and smoother path than the other four basic heuristic algorithms, such as the ACO, MRFO, WOA and SSA algorithm. Because many advanced strategies, such as Cauchy inverse learning, the sine–cosine algorithm and the Lévy flight strategy are added to the proposed ISSA algorithm, the proposed ISSA algorithm is more competitive than the other four basic heuristic algorithms.

**Figure 7:**
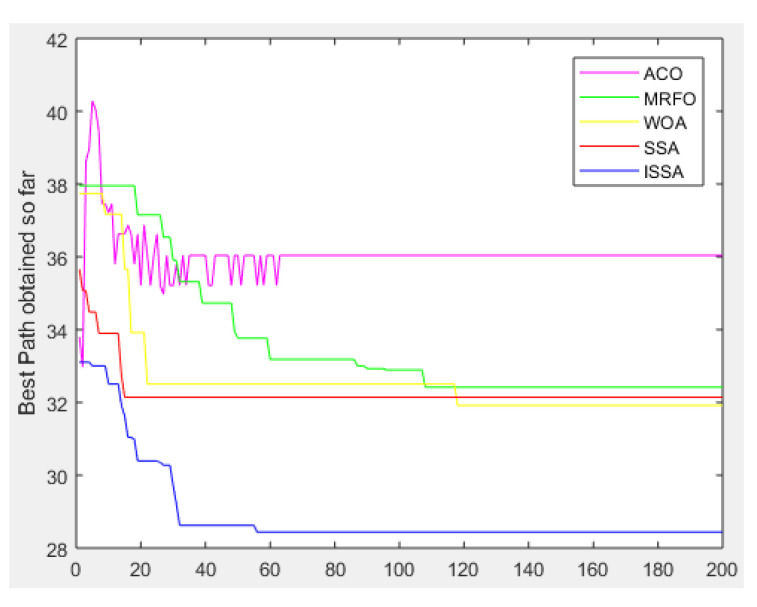
Convergence curve of the algorithms (ACO, MRFO, WOA, SSA, ISSA) in environment model 1.

Figure 7 shows the convergence of the proposed ISSA algorithm and the other four basic heuristic algorithms in solving the global optimal path problem in environmental model 1. In addition, the purple solid line represents the ACO algorithm, the green solid line represents the MRFO algorithm, the yellow solid line represents the WOA algorithm, the red solid line represents the SSA algorithm and the blue solid line represents the proposed ISSA algorithm.

As can be seen from the convergence curve plot, with an increase in iteration times, the advantages of the proposed ISSA algorithm are more obvious. The convergence speed of the proposed ISSA algorithm is faster than the other four basic heuristic algorithms from the beginning, and the final convergence accuracy of the proposed ISSA algorithm is higher than that of the other four basic heuristic algorithms.

**Figure 8:**
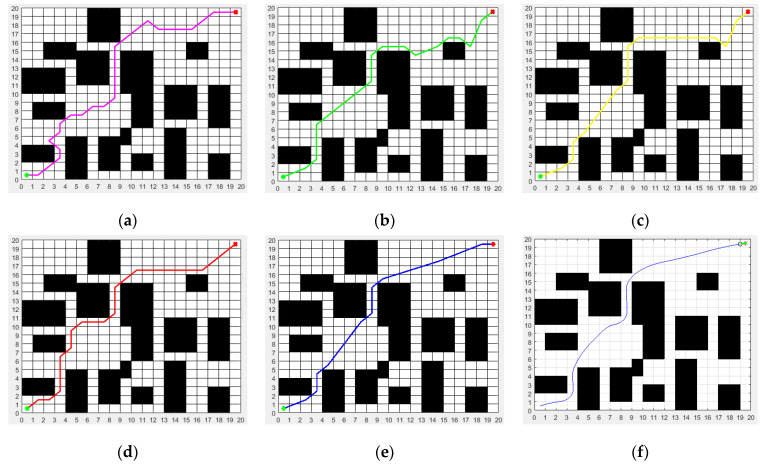
Path planned by the algorithms (ACO, MRFO, WOA, SSA, ISSA and ISSA-DWA) in environment model 2: (**a**) path planned by ACO; (**b**) path planned by MRFO; (**c**) path planned by WOA; (**d**) path planned by SSA; (**e**) path planned by ISSA; (**f**) path planned by ISSA-DWA. The green dot represents the start point and the red dot represents the end point.

In order to further verify the performance of the proposed ISSA algorithm, the second group of static obstacle avoidance simulation experiments was carried out by selecting environment model 2, which has a more complex obstacle distribution, instead of environment model 1. In environment model 2, the proposed ISSA algorithm and the other four heuristic algorithms, such as the ACO, MRFO, WOA and SSA, were applied to the static path planning of mobile robot, and the proposed ISSA-DWA algorithm was applied to the dynamic path planning of the mobile robot. The simulation results of the second group of experiments are shown in Figure 8.

Figure 8a–e show the static path planning and static obstacle avoidance effects of the ACO, MRFO, WOA, SSA and ISSA in environmental model 2, respectively, and Figure 8f shows the dynamic path planning and static obstacle avoidance effects of the proposed ISSA-DWA algorithm in environmental model 2.

**Figure 9:**
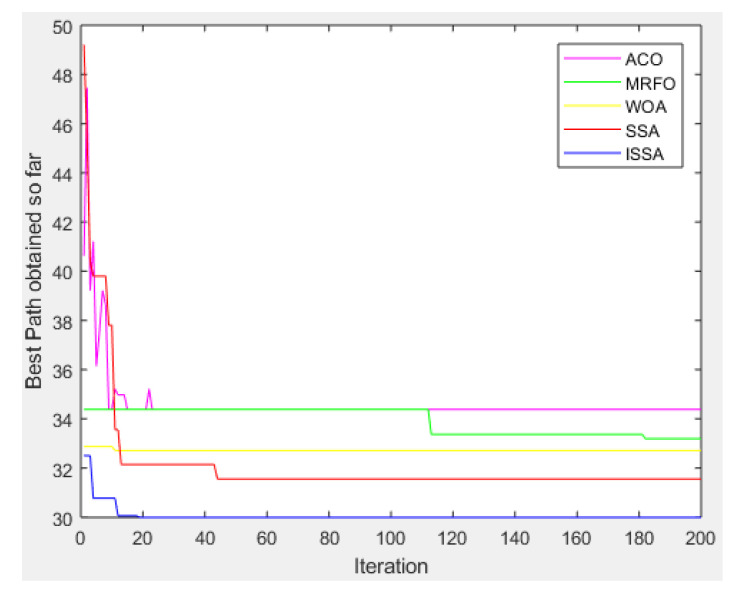
Convergence curve of the algorithms (ACO, MRFO, WOA, SSA, ISSA) in environment model 2.

Figure 9 shows the convergence of the proposed ISSA algorithm and the other four basic heuristic algorithms in solving the global optimal path problem in environmental model 2.

As can be seen from the curve chart, in the complex environment model 2, with an increase in iteration times, the proposed ISSA algorithm has an excellent performance. Regarding the convergence speed, the MRFO algorithm is the slowest and the proposed ISSA algorithm is the fastest. Regarding the convergence accuracy, the ACO algorithm is the worst and the proposed ISSA algorithm is the best. Obviously, considering the convergence speed and convergence accuracy, the convergence speed of the ISSA algorithm is faster than that of the other four basic heuristic algorithms, and the final convergence accuracy is also higher than that of the other four basic heuristic algorithms.

**Figure 10:**
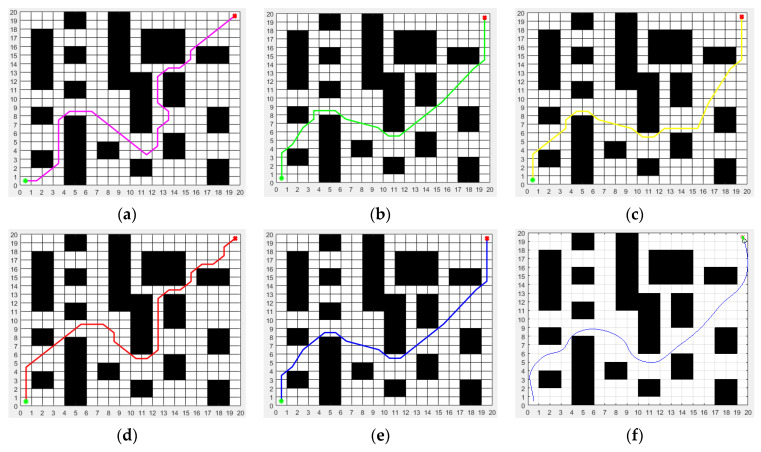
Path planned by the algorithms (ACO, MRFO, WOA, SSA, ISSA and ISSA-DWA) in environment 3: (**a**) path planned by ACO; (**b**) path planned by MRFO; (**c**) path planned by WOA; (**d**) path planned by SSA; (**e**) path planned by ISSA; (**f**) path planned by ISSA-DWA. The green dot represents the start point and the red dot represents the end point.

To verify the generalization of the proposed ISSA algorithm, the third group of static obstacle avoidance simulation experiments was carried out by selecting environment model 3, which has a more complicated obstacle distribution than environment model 1 and environment model 2. In environmental model 3, the proposed ISSA algorithm and four other heuristic algorithms, such as the ACO, MRFO, WOA and SSA, were applied to the static path planning of the mobile robot, and the proposed ISSA-DWA algorithm was applied to the dynamic path planning of the mobile robot. The simulation results of the third group of experiments are shown in Figure 10.

Figure 10a–e show the static path planning and static obstacle avoidance effects of the ACO, MRFO, WOA, SSA and ISSA algorithm in environmental model 3, respectively. Figure 10f shows the dynamic path planning and static obstacle avoidance effects of the proposed ISSA-DWA algorithm in environmental model 3.

**Figure 11:**
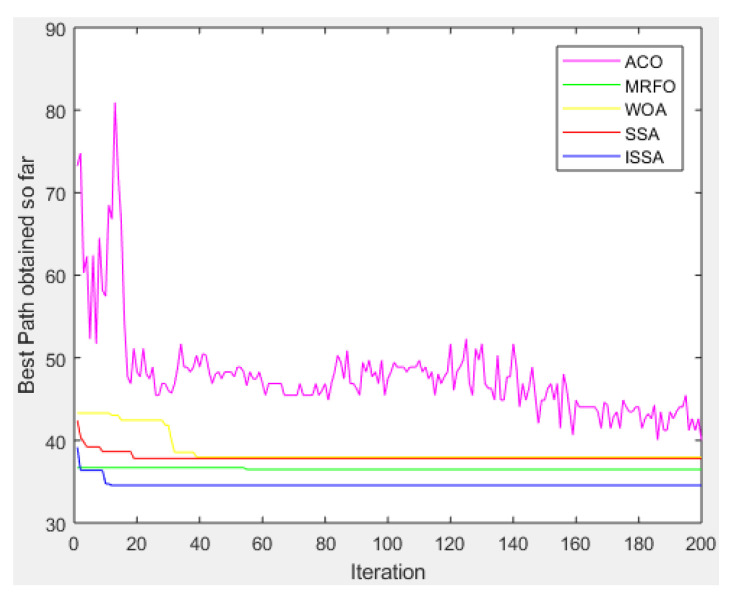
Convergence curve of the algorithms (ACO, MRFO, WOA, SSA, ISSA) in environment model 3.

Figure 11 shows the convergence of the proposed ISSA algorithm and the other four basic heuristic algorithms in solving the global optimal path problem in environmental model 3.

As can be seen from the figure, when increasing the complexity of the obstacle distribution, the excellent performance of the proposed ISSA algorithm is more apparent. In environmental model 3, when the convergence speed and convergence accuracy are considered as the measurement criteria, with an increase in iteration times, the convergence speed of the algorithms can be ranked from fast to slow as ISSA, SSA, WOA, MRFO and ACO. The convergence accuracy of the algorithms can be ranked from high to low as the proposed ISSA, MRFO, SSA, WOA and ACO. Therefore, through a series of experiments, such as environment model 1, environment model 2 and environment model 3, the convergence performance of the proposed ISSA algorithm is the best, which also proves that the ISSA algorithm proposed in this paper is not only effective in improving the standard SSA algorithm but also superior to the other four basic heuristic algorithms.

After completing the three groups of static obstacle comparison experiments, the performance of the ACO, MRFO, WOA, SSA and ISSA algorithm should be summarized in digital form. It is assumed that the path planned by the algorithms can be described as a set of the path points, and then the continuous turning angles between the path points $P_{s}$ and $P_{s+1}$ (*s* = 1, 2, …, *m*) within the path *P* are denoted as $\theta_{s}$. In order to further evaluate the smoothness of the path planned by the above proposed algorithms, a smoothness index called *smt* is defined and expressed by the following formula:

(20) $$smt=\sum_{s=0}^{m}\frac{\pi \cdot abs(\pi -\theta_{s+1}+\theta_{s})}{180\cdot (\pi +\theta_{s+1}+\theta_{s})}$$

where *m* is the number of path points of the set *P*, and $\theta_{s+1}$ is the next turning angle of $\theta_{s}$. The smaller the *smt*, the smoother the path.

In summary, the results of the three groups of static obstacle avoidance show that the proposed ISSA-DWA algorithm can plan a smooth global optimal path in environmental model 1, environmental model 2 and environmental model 3 safely and efficiently, no matter how the obstacle distribution changes. Since the proposed ISSA-DWA algorithm adds its dynamic characteristics on the basis of the proposed ISSA algorithm, when only the static characteristics of path planning are considered, it can be regarded that the effect of the proposed ISSA-DWA is equivalent to that of the proposed ISSA. Therefore, when summarizing the results of the three groups of static obstacle avoidance comparative experiments in digital form, it is only necessary to compare the performance of the proposed ISSA with the other algorithms, such as the ACO, MRFO, WOA and SSA. The algorithm performance indexes of the three groups of static simulation experiments are shown in Table 1.

### 5.3. Dynamic Obstacle Avoidance Experiment

**Figure 12:**
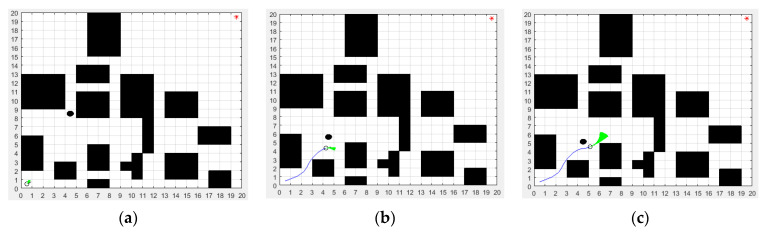
Local dynamic obstacle avoidance effect of ISSA-DWA in environment model 1: (**a**) the initial state; (**b**) the avoiding state; (**c**) the successful state.

In the improved DWA section, the maximum linear velocity and maximum angular velocity were set to 3.6 m/s and 45 rad/s, respectively. The maximum linear acceleration and the maximum angular acceleration were set to 0.35 m/s^2^ and 80 rad/s^2^, respectively. The linear velocity resolution was 0.01 m/s. The angular velocity resolution was 1 rad/s. The four weighting factors α,β,γ,φ of the evaluation function were set to 0.06, 0.2, 0.1 and 0.8, respectively. In order to verify the dynamic obstacle avoidance ability of the proposed ISSA-DWA algorithm, three groups of dynamic obstacle avoidance simulation experiments were set up in this paper. By adding black dynamic obstacles on the basis of environment model 1, environment model 2 and environment model 3, the proposed ISSA-DWA algorithm was used to avoid the black dynamic obstacle in the above three environment models. The results of the first group of dynamic obstacle avoidance experiments in environmental model 1 are shown in Figure 12.

Figure 12a shows the initial state of the mobile robot and black dynamic obstacle. Figure 12b shows the state where the mobile robot is avoiding the black dynamic obstacle. Figure 12c shows the state where the mobile robot successfully avoids the black dynamic obstacle. As can be seen from the figure, in the initial state, the start position of the mobile robot is the coordinate (1,1) and the start position of the black dynamic obstacle is the coordinate (5,9). In the avoiding state, the mobile robot moves along the global optimal path planned by the ISSA algorithm, and the black dynamic obstacle moves from top to bottom at a speed of 0.2 m/s. When the mobile robot encounters the black dynamic obstacle, it first makes an avoidance action in the direction of approximately 20 degrees to the lower right, and actively winds around the upper part of the black dynamic obstacle. After overtaking the black dynamic obstacle, the mobile robot continues to move toward the target point in the direction of approximately 42 degrees to the upper right, thus completing the whole avoidance process of the black dynamic obstacle.

**Figure 13:**
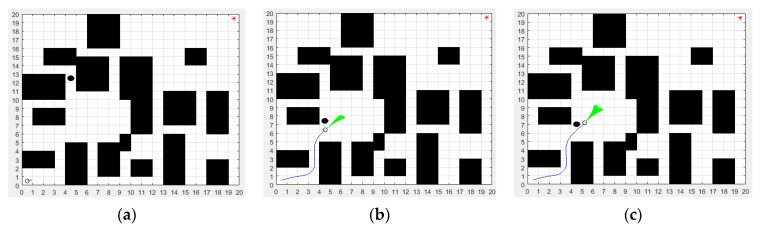
Local dynamic obstacle avoidance effect of ISSA-DWA in environment model 2: (**a**) the initial state; (**b**) the avoiding state; (**c**) the successful state.

To further verify the dynamic obstacle avoidance ability of the proposed ISSA-DWA, the second group of dynamic obstacle avoidance simulation experiments was carried out in environmental model 2, and the experimental results are shown in Figure 13.

Figure 13a shows the initial state of the mobile robot and black dynamic obstacle. Figure 13b shows the state where the mobile robot is avoiding the black dynamic obstacle. Figure 13c shows the state where the mobile robot successfully avoids the black dynamic obstacle. As can be seen from the figure, in the initial state, the start position of the mobile robot is the coordinate (1,1) and the start position of the black dynamic obstacle is the coordinate (5,13). In the avoiding state, the mobile robot moves along the global optimal path planned by the ISSA algorithm, and the black dynamic obstacle moves from top to bottom at a speed of 0.3 m/s. When the mobile robot encounters the black dynamic obstacle, it first makes an avoidance action in the direction of approximately 40 degrees to the upper right, and actively winds around the black dynamic obstacle. After overtaking the black dynamic obstacle, the mobile robot continues to move toward the target point in the direction of approximately 33 degrees to the upper right, thus completing the whole avoidance process of the black dynamic obstacle.

**Figure 14:**
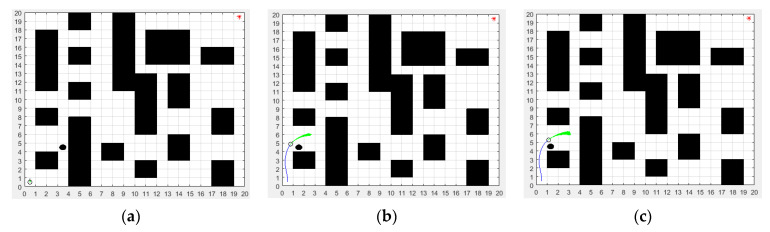
Local dynamic obstacle avoidance effect of ISSA-DWA in environment model 3: (**a**) the initial state; (**b**) the avoiding state; (**c**) the successful state.

In order to verify the excellence dynamic obstacle avoidance ability and generalization of the proposed ISSA-DWA algorithm, the third group of dynamic obstacle avoidance simulation experiments was carried out in environmental model 3, and the experimental results are shown in Figure 14.

Figure 14a shows the initial state of the mobile robot and black dynamic obstacle. Figure 14b shows the state where the mobile robot is avoiding the black dynamic obstacle. Figure 14c shows the state where the mobile robot successfully avoids the black dynamic obstacle. In the initial state, the start position of the mobile robot is the coordinate (1,1) and the start position of the black dynamic obstacle is the coordinate (4,5). In the avoiding state, the black dynamic obstacle moves from right to left at a speed of 0.25 m/s. When the mobile robot encounters the black dynamic obstacle, it first makes an evasive action in the direction of approximately 55 degrees to the upper right, and actively winds around the upper part of the black dynamic obstacle. After overtaking the black dynamic obstacle, the mobile robot continues to move toward the target point in the direction of approximately 46 degrees to the upper right, thus completing the whole evasive process of the black dynamic obstacle.

In summary, the results of the three groups of dynamic obstacle avoidance for the mobile robot show that the proposed ISSA-DWA algorithm can not only smooth the path but can also avoid dynamic obstacles in real time. In addition, this paper innovatively proposed three dynamic obstacle avoidance performance indexes, such as obstacle avoidance distance, obstacle avoidance time-consumption and obstacle avoidance angle of the mobile robot. The performance index results of the three groups of dynamic obstacle avoidance simulation experiments are summarized in Table 2.

In summary, the results of three groups of static and dynamic obstacle avoidance comparative experiments show that the proposed ISSA-DWA fusion algorithm is better than the proposed ISSA algorithm and the other four heuristic algorithms, such as the ACO, MRFO, WOA and SSA, when both static and dynamic characteristics of the algorithm are considered. It not only retains the global optimal path planning ability of the proposed ISSA algorithm but also has the ability to smooth the path and avoid dynamic obstacles in real time.

## 6. Conclusions

In this study, a novel ISSA-DWA was proposed to solve the optimal path planning problem of mobile robot. Firstly, in order to enrich the diversity of the sparrow population and avoid a premature convergence of the algorithm, the Cauchy reverse learning strategy was used to initialize the sparrow population. Secondly, the sine–cosine algorithm was used to update the producers’ position to balance the global search and local exploration capabilities of the algorithm. Furthermore, the Lévy flight strategy was used to update the scroungers’ position, which not only improves the ability of the algorithm to jump out of the local optimum, but also accelerates the convergence. Finally, the proposed ISSA algorithm and the improved DWA method were combined as a novel fusion algorithm, named ISSA-DWA algorithm. The effectiveness of the proposed ISSA-DWA was verified in the three groups of static and dynamic avoidance comparison experiments. When both static and dynamic characteristics of the algorithm are considered, the proposed ISSA-DWA’s performance on the optimal path planning problem outperforms the proposed ISSA and the other four heuristic algorithms, such as the ACO, MRFO, WOA and SSA. The experimental results demonstrate that the proposed ISSA-DWA can not only solve the shortcomings of the standard SSA but can also plan the global optimal path, which has the ability to smooth the path and avoid dynamic obstacles in real time. The proposed ISSA-DWA was only verified on the mobile robot’s simulation of the optimal path problem. In order to apply it to the practical engineering problem, future research should focus on extending it to the real mobile robot to verify the effectiveness of the proposed methods.

## Figures and Tables

**Table 1:** Comparison of performance indexes of the algorithms (ACO, MRFO, WOA, SSA and ISSA) in three environment models.

ENV. Model		Index	Path Length (m)	Turning Times	Smoothness (*smt*)	Execution Time (s)
	Algorithm					
ENV. 1	ACO		32.971	17	0.271	21.053
	MRFO		32.419	9	0.114	3.651
	WOA		31.921	11	0.345	6.966
	SSA		32.142	14	0.281	1.956
	ISSA		28.438	4	0.092	1.075
ENV. 2	ACO		34.385	11	0.337	18.633
	MRFO		33.191	13	0.144	1.957
	WOA		32.715	11	0.111	7.257
	SSA		31.556	14	0.282	2.838
	ISSA		29.992	7	0.096	1.157
ENV. 3	ACO		40.042	16	0.349	20.202
	MRFO		35.126	12	0.194	1.913
	WOA		36.792	14	0.193	6.911
	SSA		37.799	17	0.408	2.233
	ISSA		34.541	7	0.189	1.121

**Table 2:** Dynamic obstacle avoidance performance of the ISSA-DWA under three environmental models.

	ENV. Model	ENV. 1	ENV. 2	ENV. 3
Avoidance Index				
distance (m)		1.632	1.423	1.125
time-consumption (s)		6.528	4.312	4.125
angle (°)		20.125	40.523	55.684

## Data Availability

Not applicable.
